# Determinants of anaemia among women of reproductive age in South Africa: A Healthy Life Trajectories Initiative (HeLTI)

**DOI:** 10.1371/journal.pone.0283645

**Published:** 2023-03-30

**Authors:** Takana M. Silubonde, Cornelius M. Smuts, Lisa J. Ware, Glory Chidumwa, Linda Malan, Shane A. Norris

**Affiliations:** 1 Centre of Excellence for Nutrition, Faculty of Health Sciences, North-West University, Potchefstroom, South Africa; 2 SAMRC/Wits Developmental Pathways for Health Research Unit, Faculty of Health Sciences, University of the Witwatersrand, Johannesburg, South Africa; 3 DSI-NRF Centre of Excellence for Human Development, University of the Witwatersrand, Johannesburg, South Africa; 4 School of Health and Human Development, University of Southampton, Southampton, United Kingdom; University of Dhaka, BANGLADESH

## Abstract

Anaemia continues to be a persistent concern among South African women of reproductive age (WRA), yet population specific information on its determinants remains sparse. We used baseline data from the Healthy Lives Trajectory Initiative a randomised trial (n = 480) to quantify factors associated with anaemia in Soweto, South Africa aged 18–25 years. We used multivariable logistic regression to describe associations with anaemia and used structural equation modelling to assess a theoretical model, which tested three categories socioeconomic status (household asset score, education level), nutritional factors (food security, leafy green vegetable and chicken and beef consumption, iron status and vitamin A status) and biodemographic factors (parity, age at start of menarche, HIV status, contraception use, anthropometry, and inflammation status). The multiple logistic regression showed that ID (OR: 2.62, 95% CI: 1.72, 3.98), iron deficiency erythropoiesis (IDE) (OR: 1.62, 95% CI: 1.07, 2.46), and elevated CRP (OR: 1.69, 95% CI: 1.04, 2.76), increased the odds of being anaemic. SEM analysis revealed Hb was directly and positively associated with adjusted ferritin (0.0031 per mg/dL; p≤0.001), and CRP (0.015 per mg/dL; p≤0.05), and directly and negatively associated with soluble transferrin receptor sTfR (-0.042 per mg/dL; p≤0.001). While contraception use had both a direct (0.34; p≤0.05) and indirect (0.11; p≤0.01) positive association with Hb. Additionally, chicken and beef consumption had a positive indirect association with Hb concentrations (0.15; p≤0.05) through adjusted ferritin. Iron deficiency was the main anaemia risk factor in this low resource setting. However, anaemia of inflammation is present. Therefore, we suggest that in our setting, WRA anaemia control programs that include interventions to reduce ID and inflammation should be tested.

## Background

Anaemia is a major public health concern among women of reproductive age (WRA) [1]. In WRA, anaemia may result in reduced work capacity and predisposes an individual to various infections [2]. Anaemia in pregnancy has been linked to an increase in low birth weight, perinatal and neonatal mortality, and preterm birth [3]. Moreover, anaemia in pregnancy has also been associated with increased maternal mortality [4]. The World Health Organisation estimates that anaemia affects approximately 42% of pregnant women and 29.4% of non-pregnant WRA worldwide [1].

South Africa has policies in place to address anaemia and iron deficiency (ID); such as the mandatory iron fortification of maize meal and wheat flour and the *Guidelines for maternity care in South Africa* recommendation for daily iron supplementation in pregnancy [5–7]. However, the prevalence of anaemia and ID remains high. In the 2012 South African National Health and Nutrition Survey conducted among WRA (15–35 years) the prevalence of anaemia, ID, and iron deficiency anaemia (IDA) was 23%, 16% and 10% respectively. According to the 2016 South African Demographic Health Survey, 31% of WRA (15–49 years) are anaemic [8]. The above national data in conjunction with a systematic review [9] point to the fact that approximately 23%-33% of South African WRA are likely to enter pregnancy with anaemia.

The aetiology of anaemia is complex. The biological causes for anaemia as outlined include iron, other micronutrient deficiencies (vitamin A, vitamin B12 and folate) and genetic disorders [10, 11]. Vitamin A has been proposed to play a role in regulating plasma iron levels, with decreasing vitamin A levels resulting in decreasing plasma iron [12–14]. Moreover a recent systematic review on anaemia risk factors in South Africa has highlighted the link between the human immunodeficiency virus (HIV)/ tuberculosis (TB) and other comorbidities to increased risk of anaemia [15]. ID has long since been identified as the major contributor to anaemia in most low-income settings, with approximately 50% of all anaemia cases being attributed to ID [16]. As a result, and in the absence of individual data on iron status, the prevention and treatment for populations at risk of anaemia is typically directed at programs to improve iron status. However, intervening with routine iron supplementation may result in potential adverse effects in settings where individuals may already be iron replete and/or with heavy burdens of infectious disease, whereby iron supplementation may result in severe aggravation of infections [17]. Additionally, Symington *et al* in a study done in pregnant South Africa reported that iron supplementation alone may not be effective in reducing the prevalence of anaemia [18].

Furthermore, studies have highlighted other significant underlying risk factors for anaemia that are likely to contribute by either decreasing the availability and consumption of micronutrient rich foods or by increasing the risk of infectious diseases [19]. These are social and environmental risk factors such as socioeconomic status, food insecurity, education, and poor sanitation [19]. In addition, substantial to the risk of anaemia are individual characteristics in WRA such as women’s use of hormonal contraception and body mass index [20]. Several studies have shown that adiposity is associated with increased odds of inflammation [21–23]. This obesity-related inflammation has been shown to reduce iron absorption from the gut, contributing towards ID. It is the complexity of addressing all risk factors that remains challenging, at least for now, in our efforts to curb anaemia and other micronutrient deficiencies at a public health level.

Data on the risk factors for anaemia among non-pregnant WRA remain sparse in South Africa. Mchiza *et al* using the South African National Health and Nutrition Examination Survey (SANHANES-1) identified sociodemographic factors, such as age (being young), belonging to the black ethnic group, residing in households with a reported low income, as factors that fuel anemia in the country [24]. However, they did not include relevant biodemographic factors such as contraception use or parity. Moreover, iron status was not adjusted for inflammation, neither did they explore the complex interrelationships between anaemia, ID, biodemographic factors, socioeconomic factors, and nutritional factors.

Nevertheless, for South Africa to achieve the World Health Assembly Nutrition Targets of a 50% reduction in anaemia by 2025, further evidence is needed on the specific risk factors for anaemia among non-pregnant WRA within this setting. Therefore, the aim of this paper was two-fold: (i) to determine the prevalence of ID and anaemia in young women; and (ii) identify factors associated with anaemia and determine the direct and indirect relationships of socioeconomic, biodemographic and nutritional factors on anaemia.

## Methods

### Study design, setting and population

We used data from a study conducted as part of the pilot baseline study of the HeLTI trial (Healthy Lives Trajectories Initiative), a multinational trial which aims to develop and evaluate an integrated continuum of care (4 phases) starting preconception and extending through pregnancy, infancy, and childhood. The goal is to optimise women’s physical and mental health, reduce childhood obesity, the risk of non-communicable diseases, as well as improve child development [25]. The methods used in this study have been previously described in detail [26]. Data was collected from the pilot phase of the main trial and data collection took place (June2018 to July 2019) at the South African Medical Research Medical Council (SAMRC) Developmental Pathways to Health Research Unit (DPHRU), located within the Chris Hani Baragwanath Academic Hospital (CHBAH), a tertiary hospital in Soweto (Southwestern Township).

A cluster design was employed for recruitment, where each Soweto community centre was a cluster. Thirty clusters with a radius of 10 km^2^ each were identified around Soweto using churches as the midpoint of each cluster. An online search was performed using the Google search engine to locate the information of all churches in Soweto. Using street address information, geolocations of each church structure were obtained, and each church was visited by fieldworkers and verified. The latitude and longitude of the 104 churches identified and verified were then classified using k-means clustering. The church with the shortest straight-line distance to the cluster centroid was selected for inclusion in the study as it was at the centre of a cluster of churches that was maximally distant from the other churches in Soweto. An equal number of participants were recruited from two randomly selected clusters.

Generally healthy, non-pregnant WRA of African descent were recruited from Soweto, a historically disadvantaged urban area of 200 km^2^ in the city of Johannesburg, Gauteng province. Women were eligible for inclusion if they were aged 18–26 years; proficient in local languages and if they had been residing in their home in Soweto for at least 3 months. Exclusion criteria were diagnosis of type-1 diabetes; cancer or epilepsy; or not able or willing to provide written informed consent. Due to South Africa’s high prevalence of HIV infection (23.2% of women aged 15–49 years [27], women who were HIV positive were included in the study for the sample to be a better representation of the general population. As a result, HIV was self-reported and CD4 and viral load were not assessed. During the recruitment process, potential participants were visited in their homes and were informed in their home language about: (i) the objectives of the study; (ii) the use of the results; and (iii) the risks and benefits of the study. An informed consent form was supplied to potentially eligible women who were interested in being part of the study. All women in the study gave written consent.

### Ethical approval and participant consent

This study was conducted in accordance with the ethical principles laid down in the Declaration of Helsinki, and all procedures involving human participants were approved by the Human Research Ethics Committees of the North-West University, Potchefstroom (NWU-0042919-S1), and the University of the Witwatersrand, Johannesburg (M171137).

### Data collection

#### Haematological biomarkers

Venous blood samples were analysed for iron status indices ferritin and soluble transferrin receptor (sTfR) vitamin A status (retinol binding protein, (RBP) and the inflammation/infection markers C-reactive protein (CRP) and alpha-1-acid glycoprotein (AGP) using the Q-Plex™ Human Micronutrient Array (7-plex, Quansys Bioscience, Logan, UT, USA) [28]. The outcome variable of interest, ferritin concentration was adjusted for inflammation as measured via AGP and CRP concentrations using the correction factors proposed by Thurnham *et al* [29]. Participants were categorised as being iron deficient if their inflammation-adjusted plasma ferritin concentration was <15 μg/L as recommended by the World Health Organisation [30].

Haemoglobin concentrations were measured from capillary blood using a calibrated Hb 201+ HemoCue® system (HemoCue Johannesburg, South Africa). Hb values were adjusted for altitude and the point-of-care cut-off Hb <12 g/dL was used to diagnose anaemia [31]. IDA was defined as ferritin <15 μg/L and Hb <12g/Dl. Iron deficient erythropoiesis (IDE) was defined as sTfR ≥8.3 mg/L. As plasma RBP is suppressed in the presence of inflammation, RBP was also adjusted for inflammation using correction factors proposed by Thurnham *et al*. [29].

### Physical measurements

Weight (kg) and height (cm) of participants were measured to calculate body mass index: BMI = weight (kg)/ (height) ^2^. Weight was measured to the nearest 100g and height to the nearest 0.1cm. Mid-upper arm circumference (MUAC) was measured to the nearest 0.1cm using a plastic measuring tape [32]. Measurement was taken at the mid-point of the upper arm, between the acromion process and the tip of the olecranon. A MUAC ≤24cm was used to define undernutrition.

### Living conditions

Questionnaires conducted to assess sociodemographic, bio-demographic and nutrition factors were 1. sociodemographic to assess education and employment status; 2. general health which included medical and reproductive history and HIV status; 3. food insecurity and a frequency food questionnaire. Field teams visited household to assess and record the household type of residence and household density and the number of household assets. Socio-economic status was assessed using a household asset score which summed the number of assets owned in the household from the following options: TV, car, washing machine, fridge, phone, radio, microwave, cell phone, DVD/Video, DSTV (cable channel), computer, internet access and medical aid. The household asset score was based on standard measures used in the Demographic and Health Surveys household questionnaire (available at: www.measuredhs.com) and has been extensively utilised in this setting [33–35].

Food insecurity was assessed using an adapted Community Childhood Hunger Identification Project (CCHIP) index [36]. Chicken and beef consumption and leafy vegetable consumption was assessed according to the frequency of consumption during the past month. The possible responses for frequency of consumption were: ‘every day’, ‘2–4 times per week’, ‘5–6 times per week’, ‘once per week’, ‘less than once per week’, ‘never’.

### Statistical analysis

Study data were collected and managed using REDCap electronic data capture tools hosted at the University of Witwatersrand [37]. Data were tested for normality by visual inspection of Q-Q plots and histograms, and the Shapiro–Wilk test. Normally distributed data are expressed as means ± SD; non-normally distributed data are expressed as medians (interquartile range [IQR]). Descriptive analyses were used to report socioeconomic, bio-demographic, inflammation, and nutritional characteristics of the study sample. Bivariate associations between anaemia and socioeconomic, bio-demographic, inflammation and nutrition were examined. Multivariable logistic regression was used to examine the factors associated with anaemia and inflammation adjusted ID. Models were constructed with the use of block stepwise regression whereby variables were entered into the model in blocks in order of anticipated importance, WHO framework and the author’s judgement. None of the included variables showed multicollinearity, with variance inflation factors <2 for each model.

**Fig 1:**
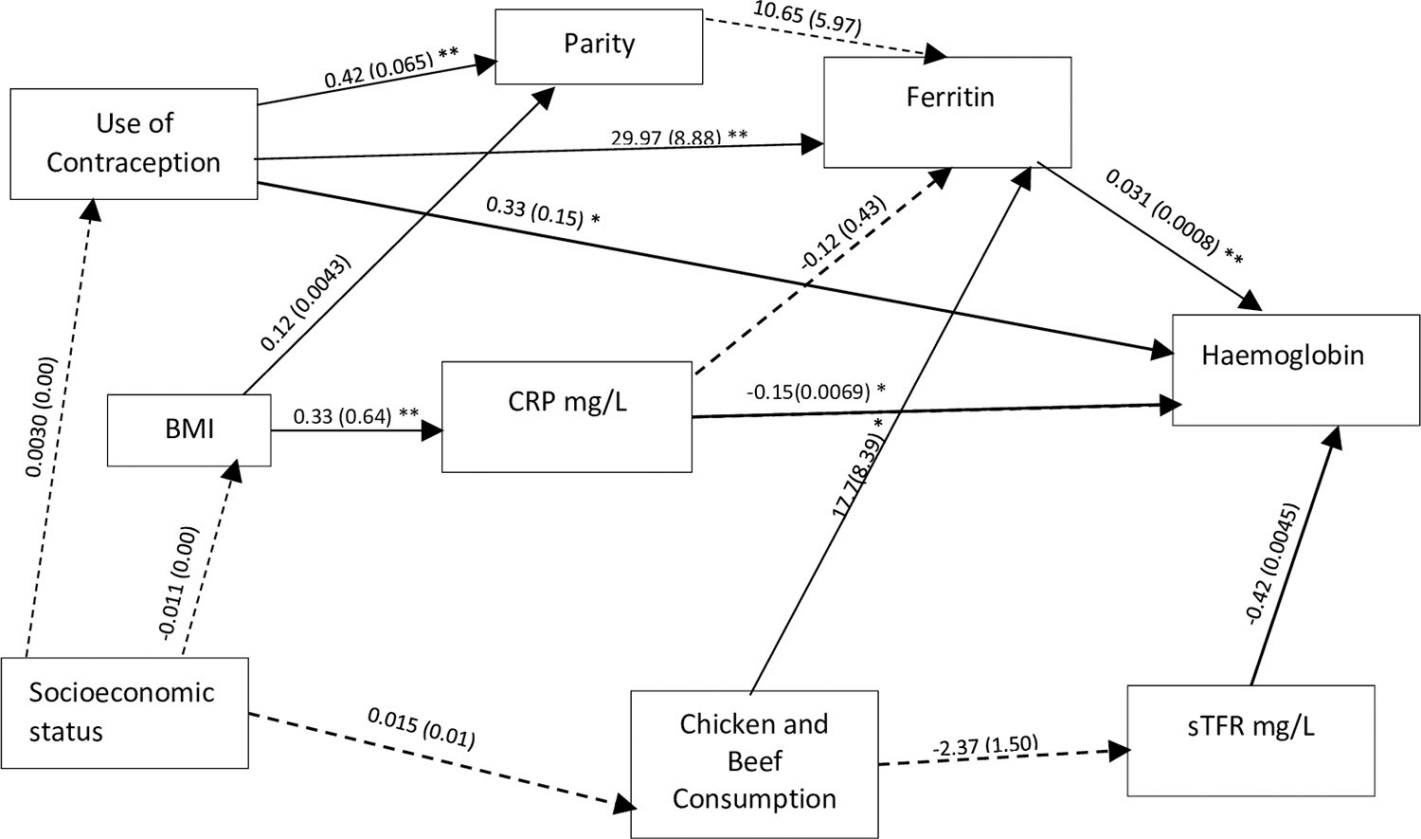
SEM framework for anaemia outcome. The solid lines represent significant direct effects and the dashed line is not a significant direct effect. All values are coefficients with standard errors in parenthesis. Significance levels: ***P<0.001, **P<0.01, *P<0.05. The model statistics were the Root Mean Square Error of Approximation for: 0.025, the Comparative Fit Index: 0.969, the Tucker-Lewis Index: 0.947 and SRMR: 0.039.

**Fig 2:**
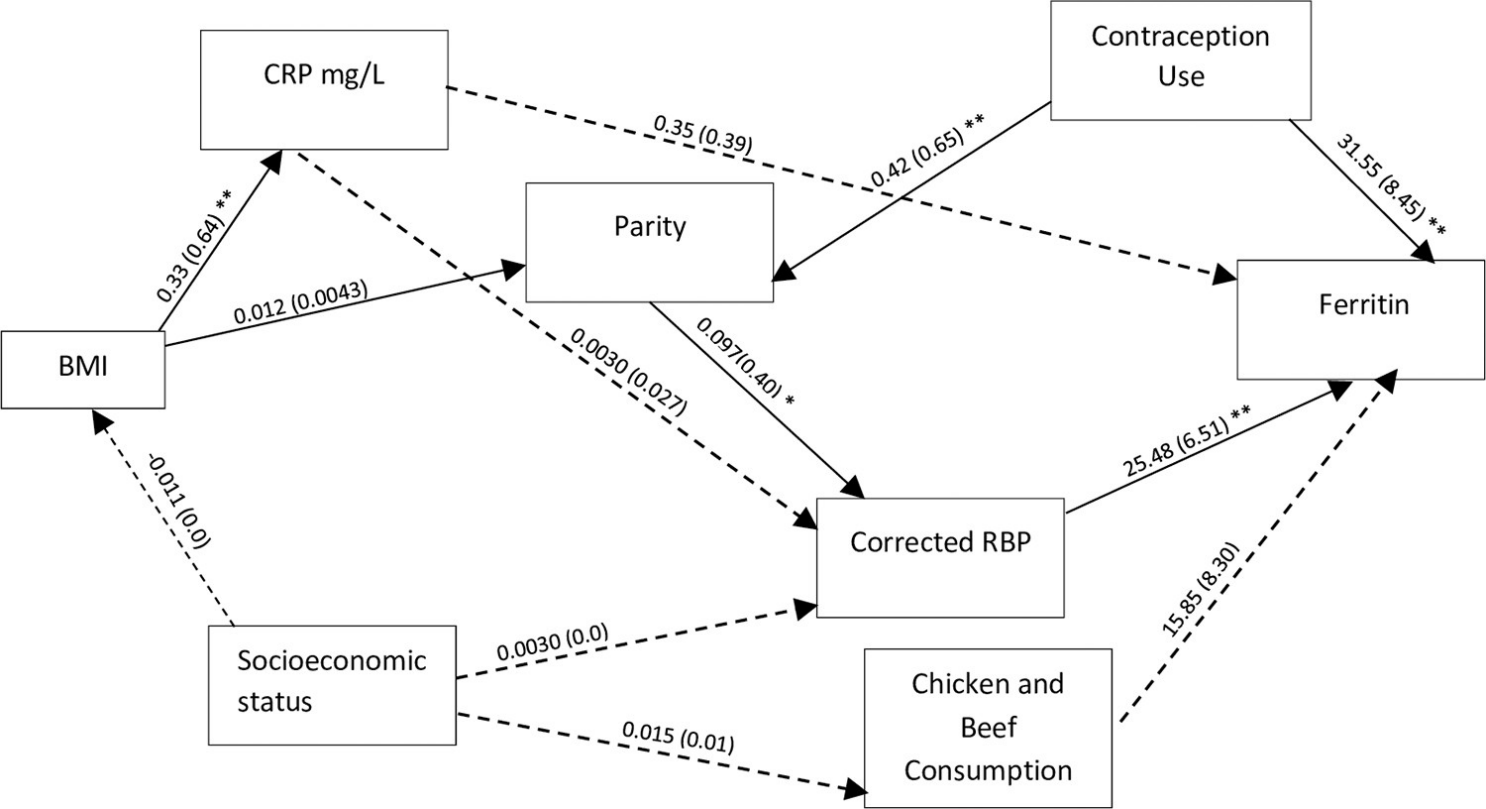
SEM framework for ID outcome. The solid lines represent significant direct effects, and the dashed line is not a significant direct effect. All values are coefficients with standard errors in parenthesis. Significance levels: ***P<0.001, **P<0.01, *P<0.05. The model statistics were the Root Mean Square Error of Approximation for: 0.026, the Comparative Fit Index: 0.958, the Tucker-Lewis Index: 0.921 and SRMR: 0.035.

Structural equation modelling was applied to examine the specific causal models and assess the comparative strength of direct and indirect relationship among independent variables with ID and anaemia (haemoglobin concentration). SEM was the analysis of choice as it allows for a pictographic representation of hypothesis-driven relationships between variables such as potential mediators, confounders, and latent variables [38]. The multivariable logistic analyses were guided by an *a priori* model (Figs 1 & 2), based on expert knowledge, literature, and results from the logistic regression. Bold lines represent statistically significant paths while dotted lines represent paths that were not statistically significant. We then hypothesised relationships *a priori* among the variables. From this framework SEM was used to estimate the associations in the different pathways between biodemographic factors, inflammation factors, sociodemographic factors, socioeconomic, nutritional factors and the two outcomes anaemia and ID. Direct, indirect, and total effects were calculated using non-linear combination estimates. To evaluate the best fitting model for the data, we reported goodness of fit indices including root mean squared error of approximation (RMSEA), comparative fit index (CFI), Tucker-Lewis index (TLI), and standardized root mean squared residual (SRMR). All data analyses were performed using Stata statistical software, version 14.1 (StataCorp, College Station, TX: Stata Corporation).

## Results

### Characteristics of women

The characteristics of women in the study sample are presented in Table 1. The median age of the participants was 21 years (IQR 19–23). Half the women were nulliparous (50.8%) and the median age at first delivery for women with children was 19 years (IQR 17–21). Most of the women had a medium household asset score (63.3%), while more than half the women (67.5%) reported consuming chicken and beef consumption at least once a week. Inflammation was present in 37.3% of the women and the mean haemoglobin concentration was 12.2 g/dL with anaemia present in 39.4% of the women. The prevalence of ID and IDA was 38.1% and 21.6% respectively and vitamin A deficiency based on RBP was observed in 3.5% of the participants.

**Table 1:** Characteristics of 18–25-year-old non pregnant women of reproductive age residing in Soweto, South Africa (n = 480).

Characteristics	Median (IQR) or n (%)
**Anaemia (altitude adjusted Hb <12g/dL)**	189 (39.4)
**ID (Inflammation adjusted < 15 μg/L)**	183 (38.1)
**IDA (Hb <12g/dL ferritin < 15 μg/L)**	104 (21.6)
**Age (y)**	21 (19.2)
**Socioeconomic**	
**Household asset score**	
**Low (1–4)**	31 (6.5)
**Medium (5–8)**	304 (63.3)
**High (>8)**	145 (30.2)
**Years of education (y)**	12 (7–12)
**Nutritional Factors**	
**At risk of food insecurity (yes)**	227 (47.3)
**Food insecure (yes)**	132 (27.5)
**Weekly consumption of leafy green vegetables(yes)**	121 (25.2)
**Weekly consumption of chicken and beef (yes)**	324 (67.5)
**Iron status biomarkers**	
Inflammation-adjusted ferritin (μg/L)^1^	25.65 (8.0–55.0)
**sTfR (mg/L)**	7.51 (5.7–10.5)
**IDE (sTfR > 8.3 mg/L)**	201 (41.9)
**Inflammation adjusted RBP μmol/L**	1.49 (1.2–1.9)
**Vitamin A status-RBP μmol/L**	
**< 0.7 deficiency**	17 (3.5)
**≥0.7–1.05 insufficiency**	59 (12.3)
**≥1.05 sufficient**	404 (84.2)
**Biodemographics**	
**Age at start of menarche (y)**	13 (12–15)
**Contraception use in the last 12 months (yes)**	332 (69.2)
**HIV positive self -reported (yes)**	21 (4.4)
**Parity**	
**nulliparous**	244(50.8)
**primiparous**	191(39.8)
**multiparous**	45(9.4)
**MUAC**	
**underweight≤24cm**	94 (19.6)
BMI (kg/m^2^)	
Underweight (<18.5 kg/m^2^)	41 (8.5)
Normal weight (18.5–24.9 kg/m^2^)	221(46.0)
Overweight (25–29.9 kg/m^2^)	104 (21.7)
Obese (≥30 kg/m^2^)	114 (23.8)
**Biomarkers of inflammation**	
**CRP (mg/L)**	1.39 (0.4–3.7)
**AGP (g/L)**	0.86 (0.7–1.0)
**Inflammatory status**	
**Inflammation (CRP > 5 mg/L and AGP > 1 g/L)**	179 (37.3)
**Incubation (CRP >5 mg/L and AGP ≤ 1 g/L)**	52 (10.8)
**Early convalescence (CRP> 5 mg/L and AGP > 1 g/L)**	43 (9.0)
**Late convalescence (CRP ≤ 5 mg/L and AGP > 1 g/L)**	84 (17.5)

Variables are either median (IQR) or n (%). MUAC, mid upper arm circumference; BMI, body mass index; AGP, α-1-acid glycoprotein; CRP, C-reactive protein; ID, iron deficiency; IDA, iron deficiency anaemia; sTfR, serum transferrin receptor; IDE, iron deficient erythropoiesis, RBP, retinol binding protein. Ferritin values were adjusted for inflammation using the correction factors suggested by Thurnham *et al*. [7]. Iron deficiency was defined as an inflammation-adjusted ferritin concentration <15mg/L. Anaemia was defined as altitude adjusted haemoglobin concentration <12g/ dL

### Multivariable analysis

Table 2 depicts the results of a multivariable logistic regression model for factors associated with ID and anaemia. There are three risk factors for anaemia among WRA: elevated CRP levels (inflammation indicator), IDE and ID. Women with elevated CRP had 1.7 greater odds for anaemia, compared to their counterparts (OR:1.7, 95% CI: 1.04, 2.8; p ≤0.05). In comparison to non-ID women, women who were ID were twice more likely to be anaemic (OR: 2.6, 95% CI: 1.7,3.4; p ≤ 0.001), while compared to their non- IDE counterparts, women who had IDE were 1.6 times more likely to be anaemic (OR:1.6, 95% CI: 1.1, 2,6; p ≤0.05).

**Table 2:** Multivariable logistic model of factors associated with anaemia and ID in non- pregnant WRA in Soweto South Africa (n = 480).

Variable	OR (95% CI)	P-value
***Anaemia outcome***		
**Iron Status biomarkers**		
**IDE (sTFR >8 .3mg/L)**	1.6 (1.1,2.5)	0.023*
**ID (inflammation- adjusted < 15 μg/L)**	2.6(1.7,3.4)	0.001*
**Inflammation markers**		
**CRP >5 mg/L**	1.7(1.04,2.8)	0.035*
**Bio-demographics**		
**Contraception use (yes)**	0.7 (0.5,1.2)	0.18
**Parity**		
**nulliparous**	ref	
**primiparous**	0.8 (0.5,1.3)	0.37
**multiparous**	0.8 (0.4,1.7)	0.61
***ID outcome***		
**RBP μmol/L**		
**RBP sufficient ≥1.05**	ref	
**RBP deficient < 0.7**	8.8 (2.6, 29.0)	<0.001*
**RBP insufficient≥0.7–1.05**	1.3 (0.7, 2.4)	0.41
**Weekly chicken and beef consumption**	0.6(0.4, 0.9)	0.001*
**Iron Status biomarkers**		
**sTFR > 8mg/L**	5.4 (3.5,8.3)	<0.001*
**Biodemographic**		
**Ever used contraception(yes)**	0.6 (0.4,1.0)	0.043*
**Parity**		
**Nulliparous**	ref	
**Primiparous**	0.8 (0.5,1.3)	0.23
**Multiparous**	0.4 (0.2,0.8)	0.014*
**Socio-economic Variables**		
**Household asset score**		
**High (>8)**	ref	
**Low (1–4)**	2.4 (1.0,5.9)	0.057
**Medium (5–8)**	0.9 (0.5,1.4)	0.50

Iron deficiency/anaemia (yes or no) was used as the outcome variable. Other variables were removed from the model because of a lack of significance. Iron deficiency was defined as an inflammation-adjusted ferritin concentration, 15mg/L. Inflammation was defined as a C-reactive protein concentration >5 mg/L or a-1-acid glycoprotein concentration >1 g/L. Vitamin A deficiency and insufficiency was defined as an adjusted retinol-binding protein or retinol concentration <0.7 μmol and <1.05 μmol, respectively. Significant results were signified with a*. Anaemia was defined as Hb < 12 g/dL. Iron deficiency erythropoiesis was defined as IDE (sTfR > 8.3 mg/L). Significant results were signified with a*.

The results show that vitamin A deficient women were more than eight times more likely to have ID (OR:8.8, 95% CI: 2.6, 29.0; p < 0.001), while IDE women were 5.4 times more likely to have ID (OR:5.4, 95% CI: 3.5, 8.3; p < 0.001). In comparison to their counterparts, women who used contraception were 0.6 times less likely to be ID (OR:0.6, 95% CI: 0.4, 1.0; p ≤ 0.05), and women who were multiparous were 0.4 times less likely to be ID (OR:0.4, 95% CI: 0.2, 0.8; p ≤ 0.01). Compared to women who did not consume chicken and beef weekly, those who consumed chicken and beef weekly were 0.6 less likely to have ID (OR:0.6, 95% CI: 0,4,0.9; p≤0.001).

### Structural equation modeling

Results from the SEM analyses for the association between biodemographic, inflammation, socio-economic and nutritional variables with anaemia are shown in Fig 1 and S1 Table. The main factors associated with Hb levels were use of contraception, corrected ferritin, weekly chicken, and beef consumption and sTfR levels. Corrected ferritin had a positive direct association with Hb (p≤0.001), sTfR also had a negative direct association with Hb concentration (p≤0.001). Chicken and beef consumption had an indirect positive association with Hb (p≤0.05), mediated by adjusted ferritin (p≤0.05). Contraception use had a positive direct (p≤0.05 and total (p≤0.05) association with Hb as well as an indirect association with Hb (p≤0.001), mediated by adjusted ferritin. CRP had a negative direct association with Hb (p≤0.05). Furthermore, BMI was directly associated with CRP (p≤0.001) as well as directly associated with parity(p≤0.001). A direct positive association was also observed between contraception use and parity (p≤0.001). As indicated by the fit statistics below in Fig 1 we infer that the hypothesized model fits the data.

The main factors associated with ferritin levels are depicted in Fig 2 and S2 Table. RBP had a direct association with corrected ferritin (p≤0.001). Contraception use had a positive direct association with ferritin levels (p ≤ 0.001) and an indirect association with ferritin levels through a pathway mediated by parity (p≤0.001) and RBP (p≤0.05). The total association of contraception use on ferritin was significant (p ≤ 0.001). While parity had a positive indirect (p≤ 0.05) association with ferritin through a pathway mediated by RBP (p≤ 0.05). Additionally, contraception use was positively associated with parity (p≤0.001) and BMI was positively associated with CRP (p≤0.001) and parity (p≤0.05). Parity had a positive and direct association with RBP (p≤ 0.05), and contraception use had a positive and direct association with RBP (p≤ 0.05) through a pathway mediated by parity (p≤0.001). As indicated by the fit statistics below in Fig 2, we infer that the hypothesized model fits the data.

## Discussion

This study was conducted to understand the associations and interactions of socioeconomic, biodemographic, nutritional and inflammation factors on anaemia among non-pregnant WRA in Soweto, South Africa. The prevalence of anaemia, ID and IDA was 39.4%, 38.1% and 21.6% respectively. From this it is evident that ID is the main contributor to anaemia, as 54.8% of anaemia is a result of ID. This result agrees with the established approximation that 50% of anaemia cases are due to ID. This approximation has been supported by studies by the World Health Organisation and Stevens *et al*, who showed that approximately 50% of anaemia was amenable to iron supplementation [4, 39]. Moreover, recent data from a meta-analysis on iron fortification concluded that ID was the main contributor to anaemia in many geographical settings [40]. However, some studies have shown that in areas of high infection burden, inflammation is the main contributor to anaemia [41, 42]. For example, a study done in Sierra Leone, a Sub-Saharan country with high inflammation, showed that 45% of non-pregnant WRA were anaemic but the driver of anaemia was not ID but inflammation [43]. In spite of the low self-reported HIV positive status reported in this study, South Africa is grappling with a high infection burden because of the tuberculosis/HIV pandemic that is concurrently occurring with a rise in non-communicable disease and obesity [44, 45]. Confirming this, 37.3% of the WRA in this study had elevated inflammation. We, therefore, expected the prevalence of IDA to be lower and for anaemia of inflammation to play a more dominant role. In contrast, the results show ID was the major contributor to anaemia. Further analysis with multivariable regression model confirmed that IDE and ID were significant risk factors for anaemia. SEM results also showed that low iron stores were associated with low haemoglobin levels. Nevertheless, the logistic regression results also revealed that the presence of inflammation, denoted by CRP, also significantly increased the risk of anaemia. Additionally, the SEM analysis showed a direct association between elevated CRP and low haemoglobin concentration. This indicates that although ID is the main risk factor, anaemia of inflammation is present. This finding highlights the importance of measuring factors other than ID when assessing the aetiology of anaemia, because when anaemia of inflammation is present, intervening with routine iron supplementation may result in severe aggravation of infections [17]. Of particular concern is evidence from an iron and folic acid randomised trial in Tanzania [46], where the trial was prematurely stopped because of excess hospitalisation associated with iron supplementation. Reinforcing this concern Oppenheimer *et al* reported that iron supplementation can increase rates of infectious diseases [17]. To combat this micronutrient powders or the consumption of biofortified foods, which have different absorption characteristics from iron supplements may be of merit, as they may not aggravate infections.

In addition to identifying the primary contributors to anaemia among WRA, using SEM allowed us to identify some entry points for interventions to reduce anaemia among women. The SEM results revealed that contraception use was directly associated with higher haemoglobin concentrations and indirectly affected haemoglobin levels through its protective effect on ferritin levels [47, 48]. Women who used contraception in this study had higher ferritin levels. It is important to note that the majority of the WRA in the study were using hormonal contraceptives and hormonal contraception use is reported to cause less bleeding during menstruation, which in turn results in less blood being lost in menstruation [49, 50]. Supporting this, a study conducted in multiple countries reported that those who used hormonal contraceptives had higher ferritin and Hb levels than those who did not [48]. Furthermore, studies done in Tanzania and Ethiopia showed that hormonal contraceptive use was associated with reduced risk in anaemia [51, 52]. Not surprisingly, contraception use in this study was higher among women who had more children, possibly resulting in these multiparous women being protected against ID.

South Africa, like other low- and middle-income countries is undergoing a nutrition transition characterised by increased consumption of processed food which is high in fat, salt and sugar foods and drinks [53–55]. Many citizens are food insecure; and as a result, processed foods which are cheaper and require less preparation have become more attractive and accessible to individuals [53]. In our study three quarters of the participants either reported being at risk of food insecurity or being food insecure. Kehoe *et al* in a study conducted in the same setting, reported the detrimental association between food insecurity and poor diet quality [33]. Hence, it is therefore not surprising that more than a quarter of the participants reported not consuming chicken and beef weekly which is high in bioavailable iron. Similarly, less than half consumed leafy vegetables. Though no association was found with leafy green vegetables and anaemia, the SEM analyses showed that weekly chicken and beef consumption, was indirectly protective against anaemia through its positive association with ferritin. As expected, chicken and beef consumption is protective against anaemia. Similar results were reported by Mchiza *et al* in their study [24].

The SEM analyses further revealed that low vitamin A stores are strongly associated with low iron levels. Vitamin A deficiency was associated with a more than eight-fold increased risk in being ID. A physiological interdependence between vitamin A deficiency and low iron status has been documented [56]. Vitamin A has been proposed to play a role in regulating plasma iron levels, with decreasing vitamin A levels resulting in decreasing plasma iron [12, 13]. Some studies have shown that combined vitamin A and iron supplementation was effective in improving ID [56–58]. Hence, iron supplementation alone, may not be adequate to deal with anaemia and ID in WRA [59]. Acknowledging this, the WHO has suggested that in areas of high prevalence of nutritional deficiencies multiple micronutrient supplements should replace iron [60].

The SEM analysis also highlights how women with high a BMI are more likely to have elevated inflammation. Several studies have shown that adiposity is associated with increased odds of inflammation [22, 23]. In a systematic review, seven out of nine countries reported that obesity was associated with elevated CRP [21]. Furthermore, obesity is a state of low-grade systemic inflammation reflected by increased concentrations of pro-inflammatory cytokines and inflammatory markers [61]. This obesity-related inflammation has been shown to reduce iron absorption from the gut, contributing towards ID. Several epidemiological studies have reported increased risk for ID in overweight and obese individuals [62–64]. Yet, the SEM analyses showed no significant direct or indirect association of obesity with ID but showed an indirect pathway/association via CRP.

The results show a relationship with BMI and parity with high BMI being associated with high parity; and women with higher parity being more likely to have higher ferritin and higher RBP levels. Research done by Abrams *et al* showed that child bearing is associated with weight gain [65]. During pregnancy women gain weight and adopt eating habits which they are unable to lose postpartum [66, 67]. This observation may indicate that the diet of multiparous WRA in this study could be richer in vitamin A and iron than that of their normal weight counterparts. This will, however, need to be further investigated.

Strengths of the study include the use of several iron biomarkers, the use of both AGP and CRP as inflammation markers as well as the correction of ferritin for the presence of inflammation. This study also made use of SEM an analytical approach which allows for simultaneous testing of multiple mediation pathways thereby avoiding the potential bias arising from neglecting the correlation between mediators. Though the analysis identified potential risk factors of anaemia and ID, yet because this is a cross sectional study, we cannot establish causality between any exposure of interest.

## Conclusions

In conclusion our results, in agreement with other low- and middle-income countries, show that iron deficiency is the main risk factor of anaemia. However, the results also highlight that anaemia of inflammation is present. Additionally, our results highlighted the possible role that vitamin A status may play in regulating iron stores. Thus, in low resource settings, with high infection burden, the use of micronutrient powders or biofortified foods, which have vitamin A and possess different absorption characteristics from iron supplements may be of merit, as they may not aggravate infections.

## Supporting information

S1 Table Direct and indirect associations of socioeconomic, bio-demographic, inflammation, and nutritional characteristics with haemoglobin concentration in women of reproductive age. (DOCX)

S2 Table Direct and indirect associations of socioeconomic, bio-demographic, inflammation, and nutritional characteristics with ferritin concentration in women of reproductive age. (DOCX)

## Abbreviations

WRA: women of reproductive age
AGP: alpha-1-acid glycoprotein
CRP: C-reactive protein
MUAC: mid upper arm circumference
HAS: household asset score
sTfR: soluble transferrin receptor
SEM: structural equation modelling
ID: iron deficiency
IDA: iron deficiency anaemia
BMI: body mass index
IDE: iron deficiency erythropoiesis
Hb: haemoglobin
HIV: human immune virus
HeLTI: Healthy Life Trajectories Initiative
