# Supplementary material for: Determinants of anaemia among women of reproductive age in South Africa: A Healthy Life Trajectories Initiative (HeLTI)
Source: PLoS One. 2023 Mar 30;18(3):e0283645. doi: 10.1371/journal.pone.0283645 (PMC10062540; doi:10.1371/journal.pone.0283645)
Supplement: S1 Table — (DOCX) [file pone.0283645.s001.docx]

**Table 1 Direct and indirect associations of socioeconomic, bio-demographic, inflammation, and nutritional characteristics with haemoglobin concentration in women of reproductive age.**

|  | **Coefficient (SE)** | **Hb** | **Ferritin** | **sTfR** | **CRP** | **HAS** | **Parity** | **Chicken & beef consumption** | **BMI** |
| --- | --- | --- | --- | --- | --- | --- | --- | --- | --- |
|  | **Variable** |  |  |  |  |  |  |  |  |
| **Ferritin** | **Direct** | 0.003*** |  |  |  |  |  |  |  |
|  | **Total** | 0.003*** |  |  |  |  |  |  |  |
| **sTfR** | **Direct** | -0.04*** |  |  |  |  |  |  |  |
|  | **Total** | -0.04*** |  |  |  |  |  |  |  |
| **Chicken& beef** | **Direct** |  | 17.8* | -2.3 |  |  |  |  |  |
|  | **Indirect** | 0.2* |  |  |  |  |  |  |  |
|  | **Total** | 0.2* | 17,8* | -2.4 |  |  |  |  |  |
| **CRP** | **Direct** | -0.02* | 0.06 |  |  |  |  |  |  |
|  | **Indirect** | 0.001 |  | -0.04 | |  |  |  |  |
|  | **Total** | -0.1* | 0.06 | -0.04 | |  |  |  |  |
| **HAS** | **Direct** |  |  |  |  |  |  | 0.02 | -0.01 |
|  | **Indirect** | 0.004 | 0.4 |  |  |  | 0.001 |  |  |
|  | **Total** | 0.004 | 0.4 |  |  | | 0.001 | 0.02 | -0.01 |
| **Contraception** | **Direct** | 0.3* | 30.00*** |  |  | 0.003 | 0.4*** |  |  |
|  | **Indirect** | 0.1** | 4.5 |  |  |  |  |  |  |
|  | **Total** | 0.5* | 34.5** |  |  | 0.003 | 0.4*** |  |  |
| **Parity** | **Direct** |  | 10.7 |  |  |  |  |  |  |
|  | **Indirect** | 0.03 |  |  |  |  |  |  |  |
|  | **Total** | 0.03 | 10.7 |  |  |  |  |  |  |
| **BMI** | **Direct** |  |  |  | 0.3*** |  | 0.01** |  |  |
|  | **Indirect** | -0.004 | 0.1 |  |  |  |  |  |  |
|  | **Total** | -0.004 | 0.1 |  | 0.3*** |  | 0.01** |  |  |

All values are linear regression coefficients. Significance levels: ***P≤0.001, **P≤0.01, *P≤0.05. Abbreviations: sTfR, serum transferrin receptor; CRP, C-reactive protein; HAS, household asset score; BMI, body mass index. Ferritin was adjusted for inflammation. The model statistics were the Root Mean Square Error of Approximation for :0.025, the Comparative Fit Index: 0.969, the Tucker-Lewis Index: 0.947 and SRMR :0.039.
