# Supplementary material for: Determinants of anaemia among women of reproductive age in South Africa: A Healthy Life Trajectories Initiative (HeLTI)
Source: PLoS One. 2023 Mar 30;18(3):e0283645. doi: 10.1371/journal.pone.0283645 (PMC10062540; doi:10.1371/journal.pone.0283645)
Supplement: S2 Table — (DOCX) [file pone.0283645.s002.docx]

**S Table 2 : Direct and indirect associations of socioeconomic, bio-demographic, inflammation, and nutritional characteristics with ferritin concentration in women of reproductive age.**

|  | **Coefficient (SE)** | **Ferritin** | **RBP** | **CRP** | **Chicken & beef consumption** | **Parity** | **Contraception** | **BMI** |
| --- | --- | --- | --- | --- | --- | --- | --- | --- |
|  | **Variable** |  |  |  |  |  |  |  |
| **RBP** | **Direct** | 25.5*** |  |  |  |  |  |  |
|  | **Total** | 25.5*** |  |  |  |  |  |  |
| **Chicken& beef** | **Direct** | 15.9 |  |  |  |  |  |  |
|  | **Indirect** |  |  |  |  |  |  |  |
|  | **Total** | 15.9 |  |  |  |  |  |  |
| **CRP** | **Direct** | 0.04 | 0.003 |  |  |  |  |  |
|  | **Indirect** | 0.08 |  |  | |  |  |  |
|  | **Total** | 0.1 | 0.003 |  | |  |  |  |
| **HAS** | **Direct** |  | 0.02 |  | 0.02 | 0.001 | 0.003 | -0.01 |
|  | **Indirect** | 0.7 | 0.0001 | -0.004 |  |  |  |  |
|  | **Total** | 0.7 | 0.02 | -0.004 | 0.02 | 0.001 | 0.003 | -0.01 |
| **Contraception** | **Direct** | 31.5*** |  |  |  | 0.4*** |  |  |
|  | **Indirect** | 1.0* | 0.04* |  |  |  |  |  |
|  | **Total** | 32.6*** | 0.04* |  |  | 0.4*** |  |  |
| **Parity** | **Direct** |  | 0.1*** |  |  |  |  |  |
|  | **Indirect** | 2.5* |  |  |  |  |  |  |
|  | **Total** | 2.5* | 0.1*** |  |  |  |  |  |
| **BMI** | **Direct** |  |  | 0.3*** |  | 0.1*** |  |  |
|  | **Indirect** | 0.07 | 0.002 |  |  |  |  |  |
|  | **Total** | 0.07 | 0.002 | 0.3^***^ |  | 0.1*** |  |  |

All values are linear regression coefficients. Significance levels. ***P≤0.001, **P≤0.01, *P≤0.05. Abbreviations: RBP, retinol binding protein; CRP, C-reactive protein; HAS, household asset score; BMI, body mass index. ***P≤0.001, **P≤0.01, *P≤0.05. The model statistics were the Root Mean Square Error of Approximation for :0.026, the Comparative Fit Index: 0.958, the Tucker-Lewis Index: 0.921 and SRMR: 0.035.
